# First records of the jewel beetles *Chrysobothris desmaresti* (Laporte & Gory, 1836) and *Hiperantha stempelmanni* Berg, 1889 (Coleoptera: Buprestidae) in Bolivia

**DOI:** 10.3897/BDJ.3.e4178

**Published:** 2015-03-26

**Authors:** Robert Perger, Fernando Guerra

**Affiliations:** ‡ Colección Boliviana de Fauna, La Paz, Bolivia; § Instituto de Ecología, La Paz, Bolivia

**Keywords:** Buprestidae, Chrysobothrini, Stigmoderini, Southern Bolivian Andes

## Abstract

The jewel beetle species *Chrysobothris desmaresti* (Laporte & Gory, 1836) and *Hiperantha stempelmanni* Berg, 1889, have been recorded in Bolivia for the first time. Both species were collected on xeric *Acacia* trees. As indicated by their presence on *Acacia* and previous records, both species may be endemic to the arid intermountain valleys of the Southern Bolivian and Northern Argentinean Andes as well as the Chaco lowland forests.

## Introduction

Bolivia is one of the worlds most entomologically diverse countries, which is indicated by the high species richness of butterflies (Gareca and Reichle 2006), tiger beetles (Pearson et al. 1999) and longhorned beetles (Wappes et al. 2013). However, recent studies suggest that especially the insect fauna of the southern subtropical part of Bolivia is still strongly sampling biased (e.g. Perger and Guerra 2012; Perger and Grossi 2013).

*Chrysobothris desmaresti* (Laporte & Gory, 1836) and *Hiperantha stempelmanni* Berg, 1889, are two conspicuous (the former is the largest of its genus) jewel beetle species that have been reported from the area of the Argentinean Chaco but not from Bolivia. *H. stempelmanni* occurs in the Cordoba, Mendoza, Salta, Santiago del Estero and Tucuman departments (Berg 1889; Bellamy 2008b; Barriga 2009), and *C. desmaresti* has been recorded in the Salta, Santiago del Estero, Catamarca, Cordoba and Tucuman departments (Kerremans 1892; Bellamy 2008a) as well. Habitat characteristics or host plant associations of both species have not been reported so far. In this short note, host plant associations and records for Bolivia are reported for both species for the first time.

## Taxon treatments

### Chrysobothris desmaresti

(Laporte & Gory, 1836)

#### Materials

- **Type status:** Other material. **Occurrence:** recordedBy: Robert Perger; Fernando Guerra; individualCount: 1; **Taxon:** scientificName: Chrysobothris desmaresti; scientificNameAuthorship: (Laporte & Gory, 1836); **Location:** higherGeography: South America, Bolivia, Andes, Tarija, Tariquía National Reserve, Salinas Valley; continent: South America; country: Bolivia; stateProvince: Tarija; municipality: O’Connor; locality: Salinas Valley; verbatimElevation: 1118 m; verbatimCoordinates: 21 45 19S 64 13 27W; decimalLatitude: -21.755278; decimalLongitude: -64.224167; **Identification:** identifiedBy: Chuck Bellamy; Mauricio Gigli; **Event:** samplingProtocol: beating sheet; samplingEffort: five hours; year: 2011; month: 12; day: 24; habitat: Acacia trees; **Record Level:** institutionCode: Colección Boliviana de Fauna

#### Distribution

Argentina: Catamarca, Cordoba, Salta, Santiago del Estero and Tucuman departments; Bolivia: Tarija department.

### Hiperantha stempelmanni

Berg, 1889

#### Materials

- **Type status:** Other material. **Occurrence:** recordedBy: Robert Perger; Fernando Guerra; individualCount: 15; behavior: foraging in Acacia flowers; **Taxon:** scientificName: Hiperantha stempelmanni; scientificNameAuthorship: Berg, 1889; **Location:** higherGeography: South America, Bolivia, Andes, Tarija, Tariquía National Reserve, Salinas Valley; continent: South America; country: Bolivia; stateProvince: Tarija; municipality: O’Connor; locality: Salinas Valley; verbatimElevation: 1118 m; verbatimCoordinates: 21 45 19S 64 13 27W; decimalLatitude: -21.755278; decimalLongitude: -64.224167; **Identification:** identifiedBy: Chuck Bellamy; **Event:** samplingProtocol: beating sheet; samplingEffort: five hours; year: 2011; month: 12; day: 24; habitat: Acacia trees; **Record Level:** institutionID: Colección Boliviana de Fauna

#### Distribution

Argentina: Cordoba, Salta, Santiago del Estero and Tucuman departments; Bolivia: Tarija department.

## Analysis

*Hiperantha stempelmanni* Berg, 1889 (n=15) and *Chrysobothris desmaresti* (Laporte & Gory, 1836) (n=1) (Fig. 1d) were collected with a beating sheet in trees of *Acacia* sp. and secondary vegetation in the Salinas Valley, a large alluvial fan that separates two mountain chains of the Southern Bolivian Andes (see material section for data; Fig. 1a, b).

## Discussion

There is no information about the habitat and host plant associations of both species in the literature. The lack of observation in the sub-humid Tucuman-Bolivian forests along the mountain slopes that border the study area (using the same method of sampling, albeit with greater effort, see Perger and Guerra 2013), the previously reported location data and the recorded presence in xeric *Acacia* trees suggest that both species are endemic to deciduous Chaco lowland forest of Southern Bolivia and North Argentina and may enter adjacent Inter-Andean dry valleys over deciduous vegetation.

## Supplementary Material

XML Treatment for Chrysobothris desmaresti

XML Treatment for Hiperantha stempelmanni

## Figures and Tables

**Figure 1a.**
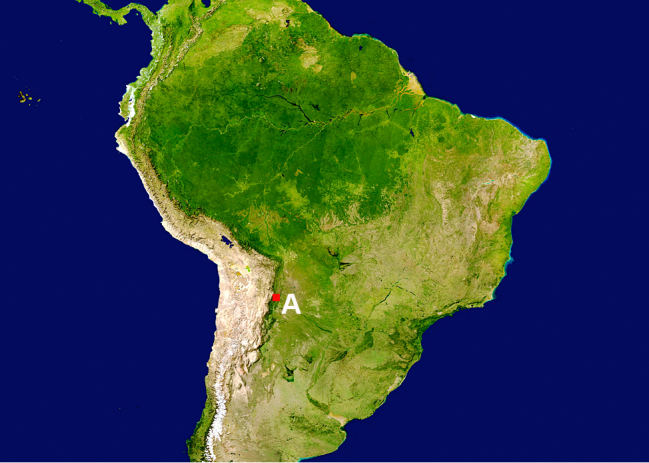
South America (NASA - U.S. Geological Survey), A study area

**Figure 1b.**
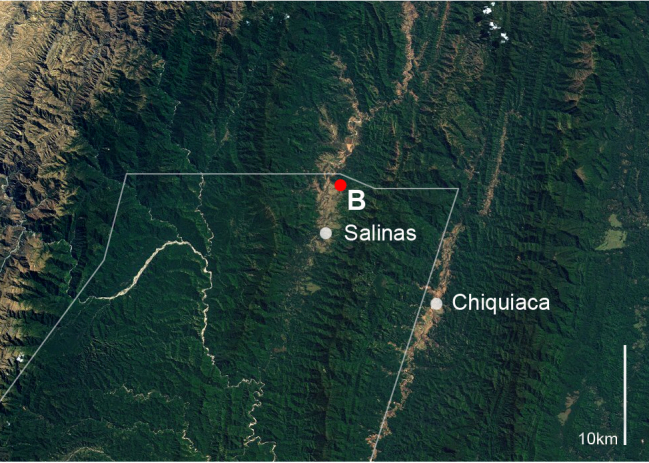
Study area (NASA - U.S. Geological Survey), Andean and Subandean area of Tarija department, Bolivia, border of Tariquía National Reserve indicated by white line; B collection location of *Chrysobothris desmaresti* (Laporte & Gory, 1836), and *Hiperantha stempelmanni* Berg, 1889

**Figure 1c.**
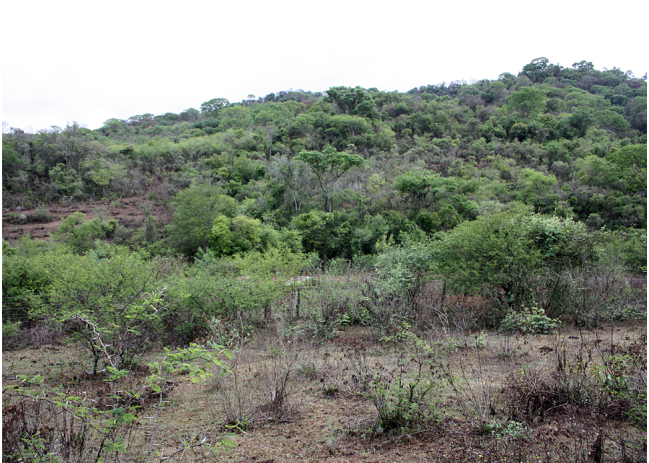
Habitat of *Chrysobothris desmaresti* (Laporte & Gory, 1836), and *Hiperantha stempelmanni* Berg, 1889

**Figure 1d.**
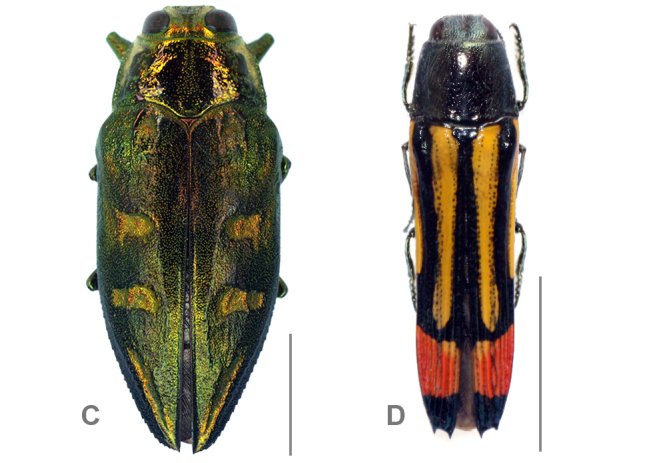
Dorsal habitus of C *Chrysobothris desmaresti* (Laporte & Gory, 1836) and D *Hiperantha stempelmanni* Berg, 1889; Scale bars = 5 mm
